# Association Between Cumulative Chemotherapy Exposure and Survival Outcomes in Advanced Biliary Tract Cancer

**DOI:** 10.3390/cancers18142263

**Published:** 2026-07-15

**Authors:** Van Khanh Nguyen, Hisashi Kosaka, Kosuke Matsui, Hideyuki Matsushima, Hidekazu Yamamoto, Gozo Kiguchi, Takuya Ohigashi, Thanh Tung Lai, Hoang Hai Duong, Kyoko Inoue, Moriyasu Takada, Hiroki Kato, Kengo Yoshii, Takashi Ito, Tsukasa Ikeura, Makoto Naganuma, Masaki Kaibori

**Affiliations:** 1 Department of Hepatobiliary Surgery, Kansai Medical University, 2-5-1 Shin-Machi, Hirakata 573-1010, Osaka, Japan; md20231204@kmu.ac.jp (V.K.N.); kosaka.his@kmu.ac.jp (H.K.); matsui.kou@kmu.ac.jp (K.M.); matsushima.hid@kmu.ac.jp (H.M.); yamamoto.hdk@kmu.ac.jp (H.Y.); kiguchi.goz@kmu.ac.jp (G.K.); ohigashi.tak@kmu.ac.jp (T.O.); laithanhtung@hmu.edu.vn (T.T.L.); md20241201@kmu.ac.jp (H.H.D.); inoue-ky@osaka-seikei.ac.jp (K.I.); m-takada@osaka-aoyama.ac.jp (M.T.); 2 Internal Gastroenterology Department, VNU University of Medicine and Pharmacy, Hanoi 100000, Vietnam; 3 Department of Surgery, Hanoi Medical University, Hanoi 100000, Vietnam; 4 Department of Mathematics and Statistics in Medical Sciences, Kyoto Prefectural University of Medicine, Kyoto City 602-8566, Kyoto, Japan; kato124@koto.kpu-m.ac.jp (H.K.); yoshii-k@koto.kpu-m.ac.jp (K.Y.); 5 Division of Gastroenterology and Hepatology, the Third Department of Internal Medicine, Kansai Medical University, 2-5-1 Shin-Machi, Hirakata 573-1010, Osaka, Japan; itou.tak@kmu.ac.jp (T.I.); ikeura.tsu@kmu.ac.jp (T.I.); naganuma@hirakata.kmu.ac.jp (M.N.)

**Keywords:** biliary tract cancer, cumulative chemotherapy exposure, overall survival, landmark analysis

## Abstract

Background: This study evaluated the association between cumulative chemotherapy exposure and overall survival (OS) in patients with advanced biliary tract cancer (BTC) and explored baseline factors associated with prolonged treatment continuation. Methods: Patients with advanced BTC receiving systemic chemotherapy were stratified by cumulative chemotherapy exposure (<8 vs. ≥8 cycles) and analyzed retrospectively. Logistic regression analysis identified baseline factors associated with receiving ≥8 chemotherapy cycles. OS was evaluated using Kaplan–Meier and landmark-adjusted time-dependent Cox proportional hazards analyses. Results: Among 86 patients analyzed, those receiving ≥8 chemotherapy cycles demonstrated more favorable inflammatory and nutritional profiles and lower metastatic burden. Median OS was longer in patients who received ≥8 chemotherapy cycles (n = 40) than in those who received <8 cycles (n = 46) (19.3 vs. 6.5 months, respectively; log-rank *p* < 0.001). Multivariable logistic regression identified metastatic disease as an independent factor associated with a lower likelihood of receiving ≥8 cycles (OR 0.34, 95% CI 0.12–0.97, *p* = 0.044). In landmark-adjusted multivariable analysis (n = 66), receiving ≥8 chemotherapy cycles remained independently associated with longer OS (HR 0.46, 95% CI 0.22–0.96, *p* = 0.039). Restricted cubic spline analysis demonstrated progressive hazard reduction with increasing chemotherapy exposure, although the magnitude of reduction attenuated beyond approximately 8 cycles. Conclusion: Greater cumulative chemotherapy exposure was associated with prolonged OS in advanced BTC, with attenuation of the survival benefit beyond approximately 8 cycles. Sustained treatment exposure may partly reflect preserved physiological reserve and ability to maintain systemic therapy in real-world clinical practice.

## Simple Summary

Biliary tract cancer is an aggressive malignancy with limited treatment options and poor long-term survival. Although systemic chemotherapy remains the standard treatment for advanced disease, the optimal duration of therapy in routine clinical practice remains uncertain. In this study, we evaluated the relationship between cumulative chemotherapy exposure and overall survival in patients with advanced biliary tract cancer treated in a real-world setting. We found that greater cumulative chemotherapy exposure was associated with longer survival, although the magnitude of survival benefit gradually diminished beyond approximately eight cycles. Patients with metastatic disease were less likely to continue treatment for prolonged periods. These findings suggest that sustained chemotherapy exposure may reflect preserved physiological reserve, favorable disease biology, and the ability of patients to tolerate and maintain systemic therapy, rather than a direct causal benefit of prolonged treatment. Our results may help clinicians individualize treatment duration and guide decisions regarding continuation of systemic therapy in patients with advanced biliary tract cancer.

## 1. Introduction

Biliary tract cancers (BTCs), which encompass intrahepatic, perihilar, and distal cholangiocarcinomas, as well as gallbladder cancer, are rare but highly aggressive malignancies associated with poor prognosis and limited long-term survival [1,2]. Although relatively uncommon, BTCs are increasing in global incidence, largely driven by the rising occurrence of extrahepatic cholangiocarcinoma [3,4]. The development of BTC is multifactorial and is associated with a variety of well-established genetic, congenital, inflammatory, and environmental risk factors [2,5].

Because clinical presentation is often nonspecific, most patients are diagnosed when their BTC is at an unresectable or metastatic stage when curative surgery is no longer feasible. Systemic chemotherapy remains the standard treatment for advanced BTC, with the combination of cisplatin and gemcitabine (GemCis) long established as the standard first-line regimen based on pivotal trials such as ABC-02 [6]. More recently, the addition of immune checkpoint inhibitors to GemCis has demonstrated additional survival benefit in the TOPAZ-1 and KEYNOTE-966 trials, and this combination has been incorporated into contemporary treatment guidelines [7,8]. Despite these advances, the optimal duration of chemotherapy balancing efficacy, cumulative toxicity, and treatment tolerability remains unclear [6,7]. Major phase III trials in advanced BTC have commonly incorporated approximately 6–8 cycles of platinum-based chemotherapy during the initial treatment phase, providing a clinically relevant benchmark; however, cumulative chemotherapy exposure in real-world practice often extends across multiple treatment lines [2,6,7,8,9]. 

In real-world clinical practice, treatment is typically continued until disease progression, unacceptable toxicity, or patient preference, resulting in substantial variability in treatment duration and cumulative chemotherapy exposure [10]. Some observational studies have suggested a potential association between prolonged chemotherapy exposure and improved survival [11,12]. However, whether prolonged chemotherapy exposure reflects true treatment benefit, favorable disease biology, or preserved physiological reserve enabling sustained therapy remains uncertain. Therefore, determinants of prolonged treatment continuation and their relationship with survival outcomes require further clarification. Moreover, the incorporation of immunotherapy into first-line treatment has further increased therapeutic heterogeneity in advanced BTC, further complicating the interpretation of cumulative chemotherapy exposure in real-world clinical practice [13].

This study aimed to evaluate the association between cumulative chemotherapy exposure and overall survival in a real-world cohort of patients with advanced BTC, while also exploring baseline factors associated with prolonged treatment continuation.

## 2. Materials and Methods

### 2.1. Patients

This retrospective study included patients aged ≥18 years with histologically or cytologically confirmed BTCs, including gallbladder cancer, intrahepatic cholangiocarcinoma, and perihilar cholangiocarcinoma. Eligible patients had received at least two cycles of systemic chemotherapy at our center between September 2018 and December 2024. Patients with mixed hepatocellular-cholangiocarcinoma, peri-ampullary cancer, or receipt of only one chemotherapy cycle were excluded.

### 2.2. Treatment

Patients received systemic chemotherapy for advanced BTC in a real-world clinical setting. Treatment selection and subsequent regimen transitions were determined based on overall clinical benefit assessment and physician judgment, considering performance status, renal function, hepatic function, and treatment tolerability. The cumulative number of chemotherapy cycles administered across all systemic treatment lines was recorded to assess the association between cumulative chemotherapy exposure and survival outcomes [10].

Treatment continuation, dose adjustment, regimen transition, and supportive interventions when clinically indicated were individualized according to clinical status, treatment tolerability, biliary complications, and physician judgment in real-world clinical practice. Various chemotherapy regimens were administered throughout the disease course according to clinical response and treatment tolerability. Chemotherapy dosing generally followed guideline-based schedules when applicable. These included gemcitabine 1000 mg/m^2^ and cisplatin 25 mg/m^2^ on days 1 and 8 of a 21-day cycle, with or without S-1 (40–60 mg/m^2^/day orally) and durvalumab 1500 mg intravenously every 3 weeks [14]. The most commonly used regimens included GemCis, with or without S-1, gemcitabine, cisplatin, and durvalumab (GCD), as well as gemcitabine or S-1 monotherapy and gemcitabine plus S-1 (GS) [7,10]. Each completed cycle of each chemotherapy regimen across all lines of systemic chemotherapy, according to its planned schedule, was counted as one chemotherapy cycle. Dose modifications and treatment delays did not alter cycle counting, whereas incomplete cycles were not counted. Durvalumab maintenance monotherapy was excluded from the cumulative chemotherapy cycle count.

### 2.3. Tumor Response Assessment

Contrast-enhanced computed tomography (CT) or magnetic resonance imaging (MRI) was generally performed every 2 months for disease monitoring. Magnetic resonance cholangiopancreatography (MRCP) or endoscopic retrograde cholangiopancreatography (ERCP) was conducted according to local clinical practice for patients with biliary obstruction or when clinically indicated.

### 2.4. Statistical Analysis

Continuous variables are presented as medians with interquartile ranges (IQRs), and categorical variables are summarized as frequencies and percentages. Group comparisons were performed using the Mann–Whitney U test for continuous variables and Pearson’s chi-square test or Fisher’s exact test for categorical variables, as appropriate.

Overall survival (OS) was defined as the time from initiation of chemotherapy to death from any cause, with patients alive at the last follow-up censored at the date of last contact. OS was estimated using the Kaplan–Meier method, and survival differences between groups were evaluated using the log-rank test. Median OS with 95% confidence interval (CI) was reported.

To minimize immortal time bias, landmark analysis was performed at 4 months. The 4-month landmark was selected to ensure adequate assessment of treatment exposure while preserving sample size. Patients surviving beyond the landmark were included in subsequent analyses.

The independent association between cumulative chemotherapy exposure and OS was evaluated using multivariable Cox proportional hazards models with cumulative chemotherapy cycles modeled as a time-dependent covariate. Patients contributed person-time to the <8-cycle category until completion of the eighth chemotherapy cycle and to the ≥8-cycle category thereafter. Potential nonlinear relationships between chemotherapy cycle number (as a continuous variable) and OS were explored using restricted cubic spline (RCS) models with three knots. Hazard ratios (HRs) with 95% CIs were reported.

The threshold of 8 cumulative chemotherapy cycles was prespecified as a clinically relevant cutoff, corresponding approximately to the standard duration of first-line chemotherapy used in the pivotal ABC-02 and TOPAZ-1 trials [6,7]. Logistic regression analysis was performed to identify baseline factors associated with receiving ≥8 cumulative chemotherapy cycles. Variables included in the multivariable model were selected based on clinical relevance and results of univariable analysis.

All statistical analyses were performed with R software, version 4.3.3 (R Foundation for Statistical Computing, Vienna, Austria). A two-sided *p* value < 0.05 was considered statistically significant.

## 3. Results

### 3.1. Baseline Characteristics

Baseline characteristics are summarized in Table 1. A total of 86 patients who received systemic chemotherapy for advanced BTC were included in the analysis and stratified according to cumulative chemotherapy exposure (<8 cycles, n = 46; ≥8 cycles, n = 40). Patients in the ≥8 cycles group had a longer median follow-up duration than those in the <8 cycles group (16.9 vs. 5.4 months, respectively). No patient proceeded to conversion surgery after systemic chemotherapy during the study period. The treatment-course characteristics of the cohort are summarized in Supplementary Table S1.

Most patients had preserved baseline performance status, with 74 (86.0%) demonstrating an Eastern Cooperative Oncology Group performance status (ECOG-PS) of 0. Metastatic disease was less frequent in the ≥8 cycles group than in the <8 cycles group (35% vs. 59%, respectively; *p* = 0.028). Patients in the ≥8 cycles group tended to have lower median neutrophil-to-lymphocyte ratios (NLRs) and C-reactive protein (CRP) levels and higher serum albumin levels compared with those in the <8 cycles group. Baseline bilirubin levels were generally comparable between groups. Overall, patients in the ≥8 cycles group demonstrated more favorable baseline inflammatory and nutritional profiles, along with lower metastatic burden.

### 3.2. Factors Associated with Receiving ≥8 Chemotherapy Cycles

Univariate and multivariable logistic regression analyses were performed to identify baseline characteristics associated with receiving ≥8 cumulative chemotherapy cycles (Table 2). In univariate analysis, higher serum albumin levels (OR 1.92, 95% CI 1.02–3.65, *p* = 0.044) were significantly associated with an increased likelihood of receiving ≥8 chemotherapy cycles, whereas higher NLRs (OR 0.85, 95% CI 0.74–0.98, *p* = 0.035) and metastatic vs. locally advanced disease (OR 0.38, 95% CI 0.16–0.91, *p* = 0.030) were associated with a lower likelihood of prolonged chemotherapy exposure.

In multivariable analysis, baseline metastatic disease remained independently associated with a lower likelihood of receiving ≥8 chemotherapy cycles (OR 0.34, 95% CI 0.12–0.97, *p* = 0.044).

### 3.3. Survival Outcomes

**Figure 1:**
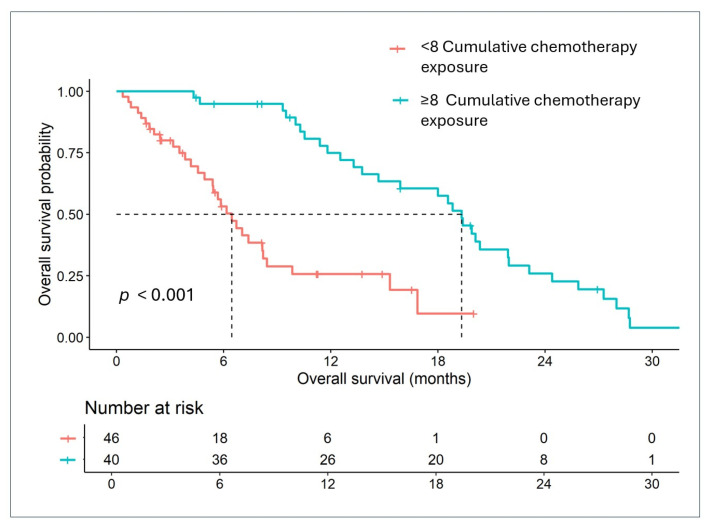
Kaplan–Meier survival curves for patients stratified by cumulative chemotherapy exposure (<8 vs. ≥8 cycles).

During the follow-up period, 61 patients died, with a median OS of 6.5 months (95% CI, 5.4–8.4) in the <8 cycles group and 19.3 months (95% CI, 14.7–22.0) in the ≥8 cycles group (log-rank *p* < 0.001). Kaplan–Meier curves demonstrated significantly prolonged OS in the ≥8 cycles group (Figure 1). In a sensitivity analysis excluding patients who received GCD as first-line therapy, Kaplan–Meier analysis demonstrated a consistent survival difference between the <8- and ≥8-cycle groups (log-rank *p* < 0.001; Supplementary Figure S1).

Factors associated with OS after landmark adjustment are summarized in Table 3. In univariate time-dependent Cox proportional hazards analysis with chemotherapy cycle number treated as a time-dependent covariate, receiving ≥8 chemotherapy cycles was significantly associated with a reduced hazard of death compared with receiving <8 cycles (HR 0.41, 95% CI 0.20–0.82, *p* = 0.012), whereas metastatic disease was associated with worse survival compared with locally advanced disease (HR 1.84, 95% CI 1.01–3.39, *p* = 0.047).

In multivariable analysis, receiving ≥8 chemotherapy cycles remained independently associated with longer OS (HR 0.46, 95% CI 0.22–0.96, *p* = 0.039; Table 3). In addition, serum albumin ≥3.5 g/dL was independently associated with longer OS (HR 0.49, 95% CI 0.24–0.99, *p* = 0.0495).

In an additional sensitivity analysis adjusting for ECOG-PS, the association between receiving ≥8 cumulative chemotherapy cycles and longer overall survival remained consistent (Supplementary Table S2).

### 3.4. Nonlinear Association Between Chemotherapy Exposure and Survival

**Figure 2:**
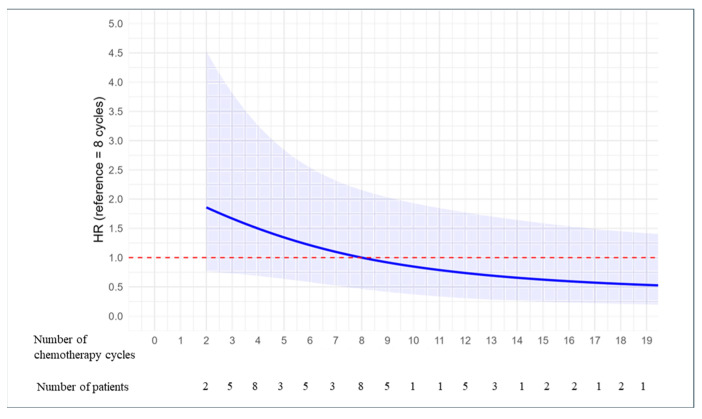
Restricted cubic spline analysis of the association between cumulative chemotherapy cycles and OS in the landmark cohort (N = 66). Reference set at 8 cycles. The central line represents the estimated hazard ratio (HR), and the blue shaded area indicates the 95% confidence interval. The red dashed horizontal line indicates the reference HR (HR = 1).

Restricted cubic spline analysis modeling cumulative chemotherapy cycles as a continuous variable demonstrated a monotonic decrease in HR with increasing cycle number. However, the magnitude of hazard reduction became less pronounced beyond approximately 7–8 cycles, suggesting attenuation of the incremental survival benefit with additional chemotherapy exposure beyond this range (Figure 2).

## 4. Discussion

This study examined the association between cumulative chemotherapy exposure and survival outcomes in patients with advanced BTC and identified baseline factors associated with treatment continuation. To our knowledge, limited real-world evidence is available regarding treatment duration and the baseline determinants of prolonged chemotherapy exposure in patients with advanced BTC. Our findings suggest that greater cumulative chemotherapy exposure was associated with prolonged OS in advanced BTC.

Previous randomized trials established the survival benefit of first-line combination chemotherapy in advanced BTC [15]. The ABC-02 trial demonstrated that GemCis administered for up to 8 cycles significantly improved OS compared with gemcitabine alone [6], thereby establishing the current standard of care [16]. However, these trials evaluated treatment within a predefined cycle range and did not fully address the survival implications of extending chemotherapy beyond this duration. In a secondary report of ABC-02, 45 patients (11%) achieved long-term survival without receiving additional chemotherapy outside the trial protocol; these long-term survivors were predominantly in the GemCis group, although no difference in quality of life was observed between treatment groups [17]. Regional studies from Asia have further supported the activity of S-1–containing regimens combined with GemCis, and more recent trials incorporating immune checkpoint inhibitors into first-line therapy have continued to reshape the therapeutic landscape [10,14,16]. Despite these advances, the clinical relevance of prolonged cumulative chemotherapy exposure remains insufficiently characterized [18,19]. Evidence supporting continuation of cytotoxic chemotherapy beyond standard treatment duration remains sparse and is largely derived from retrospective analyses, with no definitive prospective data confirming a survival advantage. In contrast, emerging maintenance-oriented strategies have also suggested that treatment de-escalation rather than indefinite continuation of cytotoxic chemotherapy may represent an alternative therapeutic approach [20]. Accordingly, in real-world practice, chemotherapy strategies frequently diverge from controlled trial settings, with variability in treatment duration and sequencing, as observed in our cohort [10,12,16,21]. In particular, first-line GemCis therapy is often limited to approximately 5 cycles in routine practice, after which alternative regimens may be considered depending on clinical response and patient status [12].

Our findings provide complementary real-world evidence indicating that preserved hepatic function may be associated with extended cumulative chemotherapy exposure. The association between cumulative chemotherapy exposure and survival strengthened with increasing treatment duration; however, the apparent incremental benefit appeared to attenuate beyond approximately 8 cycles. This finding should be interpreted cautiously because of the wide confidence intervals at the extremes of the spline curve. Findings from TOPAZ-1 and KEYNOTE-966 suggest that the incremental benefit of maintenance chemotherapy beyond the conventional induction phase may be limited, supporting a more individualized approach to treatment continuation, particularly given cumulative hematologic toxicity [7,8,9]. Previous retrospective analyses evaluating maintenance GemCis beyond the conventional induction phase have similarly reported limited or inconsistent survival benefit with prolonged chemotherapy continuation [22]. These findings suggest that cumulative chemotherapy exposure may reflect not only treatment duration itself, but also underlying tumor biology, preserved hepatic reserve, and the ability to tolerate sustained systemic therapy [23]. Therefore, prolonged chemotherapy exposure may partly reflect favorable physiological reserve and treatment tolerance rather than treatment effect alone.

In several malignancies, maintenance or prolonged chemotherapy exposure has been associated with improved disease control and survival in selected settings [24,25]. Although evidence in BTC remains limited and heterogeneous, some studies and real-world syntheses have reported improved OS with continued chemotherapy [11,21,26]. No definitive molecular mechanism has been established to explain these observations. Nonetheless, preserved hepatic reserve may represent both a prerequisite for treatment continuation and a marker of preserved physiological condition. Appropriate supportive care and biliary management when clinically indicated may further facilitate sustained systemic therapy [27,28]. In real-world management of advanced BTC, sustained systemic therapy often depends on multidisciplinary supportive care, including biliary drainage management, infection control, nutritional assessment and support, and maintenance of hepatic function according to clinical need.

From a biological perspective, adequate hepatic function and relief of biliary obstruction may facilitate drug metabolism, reduce treatment-related toxicity, and support sustained systemic therapy [27,29]. The diminishing incremental benefit observed with prolonged therapy may reflect multiple mechanisms. Patients able to receive prolonged chemotherapy may inherently exhibit more indolent or chemotherapy-sensitive tumor biology, suggesting that part of the observed survival advantage reflects favorable disease behavior rather than treatment effect alone [30]. In addition, prolonged cytotoxic exposure may contribute to therapeutic resistance through progressive clonal adaptation, while cumulative toxicity, nutritional decline, and systemic frailty may gradually counterbalance the benefit of continued treatment [30].

These findings have potential clinical implications. For patients with preserved performance status and hepatic function, continuation of chemotherapy beyond the conventional 6–8 cycles may be reasonable when treatment remains tolerable and disease control is maintained. In contrast, for patients with declining physiological reserve or emerging toxicity, dose modification, treatment de-intensification, or transition to maintenance strategies may provide a more balanced risk–benefit profile, in line with current clinical guidelines [16]. Such treatment strategies may help minimize cumulative toxicity while preserving disease control in patients unlikely to benefit from an indefinite duration of cytotoxic therapy.

Strengths of this study include the evaluation of cumulative chemotherapy exposure across the entire disease course, as well as the identification of factors associated with treatment continuation, in a real-world cohort of patients with advanced BTC. In addition, complementary landmark analysis, time-dependent Cox modeling, and restricted cubic spline analysis were applied to provide a more robust assessment of the association between cumulative chemotherapy exposure and survival outcomes. Regarding limitations, the retrospective single-center design is subject to potential selection bias and residual confounding, despite adjustment using time-dependent modeling and landmark analysis. The relatively small sample size, particularly within the landmark cohort, limits the statistical power and precision of non-linear estimates. Furthermore, treatment heterogeneity, including variation in chemotherapy regimens and limited incorporation of contemporary immunotherapy, may have introduced residual confounding and may also affect generalizability to current practice. Finally, several potentially important variables, such as molecular tumor characteristics, detailed toxicity profiles, socioeconomic status, psychological factors, and supportive care, were not available for analysis. Accordingly, these findings require validation in larger prospective multicenter studies.

## 5. Conclusions

Greater cumulative chemotherapy exposure was associated with longer OS in advanced BTC, although incremental survival benefit appeared to be attenuated beyond approximately 8 cycles. Prolonged treatment exposure may partly reflect preserved physiological reserve and the capacity to sustain systemic therapy in selected patients in real-world clinical practice. The clinical value of treatment continuation beyond 8 cycles requires further prospective evaluation.

## Figures and Tables

**Table 1:** Baseline characteristics according to cumulative chemotherapy exposure (<8 vs. ≥8 cycles).

Factor	Overall (N = 86)	Chemotherapy Cycles <8 (N = 46)	Chemotherapy Cycles ≥8 (N = 40)	*p* Value
Age ≥ 75 years	36 (42.0%)	21 (46%)	15 (38%)	0.445
Male, n (%)	48 (56.0%)	28 (61%)	20 (50%)	0.311
ECOG-PS				<0.001
0	74 (86%)	34 (74%)	40 (100%)	
1	9 (10%)	9 (20%)	0 (0%)	
2	2 (2.3%)	2 (4.3%)	0 (0%)	
3	1 (1.2%)	1 (2.2%)	0 (0%)	
BMI	21.1 [19.2, 22.4]	21.3 [19.7, 22.4]	21.0 [19.0, 22.4]	0.467
Cholangitis, n (%)	65 (76%)	34 (76%)	31 (78%)	0.832
Drainage, n (%)	49 (56.9%)	25 (54%)	24 (60%)	0.597
Tumor site n (%)				0.347
iCCA	32 (37.0%)	18 (39%)	14 (35%)	
GBC	24 (28.0%)	15 (33%)	9 (23%)	
pCCA	30 (35.0%)	13 (28%)	17 (43%)	
Metastasis, n (%)	41 (48%)	27 (59%)	14 (35%)	0.028
WBC	6350 [5100, 9400]	6450 [5100, 9600]	6250 [5050, 8300]	0.544
NLR	3.3 [2.1, 6.0]	4.1 [2.1, 7.6]	3.1 [2.0, 4.8]	0.142
PLT	26.4 [20.1, 31.5]	26.8 [20.6, 31.5]	25.8 [19.1, 31.5]	0.497
Albumin	3.3 [2.8, 3.9]	3.1 [2.7, 3.6]	3.4 [3.1, 4.0]	0.026
Bilirubin	0.7 [0.5, 1.2]	0.7 [0.5, 1.2]	0.7 [0.5, 1.2]	0.845
AST	31.5 [22.0, 50.0]	29.5 [23.0, 47.0]	33.5 [22.0, 59.0]	0.652
ALT	27.5 [17.0, 46.0]	26.0 [16.0, 39.0]	30.5 [17.0, 51.0]	0.305
CRP	1.1 [0.3, 3.2]	1.4 [0.5, 4.8]	1.0 [0.2, 1.8]	0.040
CEA (U/mL)	4.1 [1.9, 8.5]	4.2 [2.4, 21.3]	3.5 [1.5, 7.1]	0.121
CA19-9, U/mL	205.8 [30.6, 3188.2]	205.8 [31.3, 5935.7]	219.1 [30.0, 1355.6]	0.438
eGFR (mL/min/1.73 m^2^)	67.5 [54.0, 81.0]	66.0 [52.0, 81.0]	68.0 [57.5, 81.0]	0.505
Follow-up duration, months	8.9 [4.5, 16.9]	5.4 [2.5, 8.2]	16.9 [10.2, 21.9]	<0.001
First-line therapy				<0.001
GemCis (±S-1)	54 (62.7%)	20 (43.5%)	34 (85.0%)	
GCD	25 (29.1%)	20 (43.5%)	5 (12.5%)	
Others *	7 (8.1%)	6 (13.0%)	1 (2.5%)	

*: Others included gemcitabine or S-1 monotherapy, durvalumab monotherapy, and gemcitabine plus S-1. Data are presented as n (%) or median (interquartile range, Q1–Q3). Pearson’s Chi-square test; Fisher’s exact test; Mann–Whitney U test. Abbreviations: BMI, body mass index; CA19-9, carbohydrate antigen 19-9; CEA, carcinoembryonic antigen; CRP, C-reactive protein; ECOG-PS, Eastern Cooperative Oncology Group performance status; eGFR, estimated glomerular filtration rate; GBC, gallbladder cancer; GemCis, gemcitabine plus cisplatin; iCCA, intrahepatic cholangiocarcinoma; pCCA, Perihilar cholangiocarcinoma; NLR, neutrophil-to-lymphocyte ratio; PLT, platelet count; WBC, white blood cell count.

**Table 2:** Logistic regression analyses of baseline factors associated with receiving ≥8 cumulative chemotherapy cycles (N = 86).

Factor	Univariate Analysis		Multivariate Analysis	
	OR (95% CI)	*p*	OR (95% CI)	*p*
Age ≥ 75 (vs. <75) years	0.71 (0.30–1.70)	0.445	0.60 (0.22–1.66)	0.326
Gender, male	1.56 (0.66–3.67)	0.312	1.29 (0.44–3.80)	0.642
Total bilirubin (mg/dL)	0.83 (0.43–1.65)	0.612	0.75 (0.34–1.62)	0.458
Metastasis (vs. locally advanced)	0.38 (0.16–0.91)	0.030	0.34 (0.12–0.97)	0.044
Albumin (g/dL)	1.92 (1.02–3.65)	0.044	1.54 (0.64–3.68)	0.667
NLR	0.85 (0.74–0.98)	0.035	0.91 (0.77–1.10)	0.465
eGFR (mL/min/1.73 m^2^)	1.01 (0.98–1.03)	0.540	1.01 (0.99–1.04)	0.568
CEA (U/mL)	0.99 (0.98–1.01)	0.298	1.00 (0.99–1.001)	0.700

Abbreviations: CEA, carcinoembryonic antigen; CI, confidence interval; eGFR, estimated glomerular filtration rate; NLR, neutrophil-to-lymphocyte ratio; OR, odds ratio. ORs for continuous variables (albumin, total bilirubin, NLR, CEA, and eGFR) were calculated per 1-unit increase.

**Table 3:** Univariate and multivariable time-dependent Cox proportional hazards analyses of OS in the landmark cohort (N = 66).

Factor	Univariate Analysis		Multivariate Analysis	
	HR (95% CI)	*p*	HR (95% CI)	*p*
Chemotherapy cycles (≥8 vs. <8)	0.41 (0.20–0.82)	0.012	0.46 (0.22–0.96)	0.039
Albumin, g/dL (≥3.5 vs. <3.5)	0.54 (0.29–1.01)	0.051	0.49 (0.24–0.99)	0.0495
Metastasis (vs. locally advanced)	1.84 (1.01–3.39)	0.047	1.64 (0.84–3.16)	0.141
Total bilirubin, mg/dL (≥1.2 vs. <1.2)	0.68 (0.34–1.34)	0.265	0.75 (0.34–1.63)	0.569
eGFR, mL/min/1.73 m^2^ (≥60 vs. <60)	0.76 (0.41–1.42)	0.405	0.70 (0.35–1.40)	0.255
CA19-9, U/mL (≥100 vs. <100)	1.08 (0.58–2.02)	0.809	1.08 (0.55–2.12)	0.813
Cholangitis at baseline (yes vs. no)	0.83 (0.55–1.26)	0.404	0.76 (0.48–1.18)	0.219

Abbreviations: CA19-9, carbohydrate antigen 19-9; CI, confidence interval; eGFR, estimated glomerular filtration rate; HR, hazard ratio; OS, overall survival. Chemotherapy cycles were included as a time-dependent covariate in the Cox model.

## Data Availability

The data that support the findings of this study are not publicly available because of the inclusion of sensitive personal information but are available from the corresponding author upon reasonable request.

## Supplementary Materials

The following supporting information can be downloaded at: https://www.mdpi.com/article/10.3390/cancers18142263/s1: Supplementary Figure S1. Kaplan–Meier survival curves according to cumulative chemotherapy exposure after exclusion of patients who received GCD as first-line therapy; Supplementary Table S1. Sensitivity analysis: multivariable time-dependent Cox regression adjusted for ECOG-PS; Supplementary Table S2. Treatment-course characteristics of the study cohort.

## Abbreviations

The following abbreviations are used in this manuscript:

ALT	alanine aminotransferase
AST	aspartate aminotransferase
BMI	body mass index
BTC	biliary tract cancer
CA 19-9	carbohydrate antigen 19-9
CEA	carcinoembryonic antigen
CI	confidence interval
CRP	C-reactive protein
CT	computed tomography
ECOG-PS	Eastern Cooperative Oncology Group performance status
eGFR	estimated glomerular filtration rate
ERCP	endoscopic retrograde cholangiopancreatography
GBC	gallbladder cancer
GCD	gemcitabine, cisplatin, and durvalumab
GemCis	gemcitabine plus cisplatin
GS	gemcitabine plus S-1
HR	hazard ratio
ICC	intrahepatic cholangiocarcinoma
IQR	interquartile range
MRI	magnetic resonance imaging
MRCP	magnetic resonance cholangiopancreatography
NLR	neutrophil-to-lymphocyte ratio
OR	odds ratio
OS	overall survival
PLT	platelet count
RCS	restricted cubic spline
WBC	white blood cell count
