# Supplementary material for: Association Between Cumulative Chemotherapy Exposure and Survival Outcomes in Advanced Biliary Tract Cancer
Source: Cancers (Basel). 2026 Jul 15;18(14):2263. doi: 10.3390/cancers18142263 (PMC13406718; doi:10.3390/cancers18142263)
Supplement: Supplementary file 1 [file cancers-18-02263-s001.zip › cancers-4379801-supplementary.pdf]

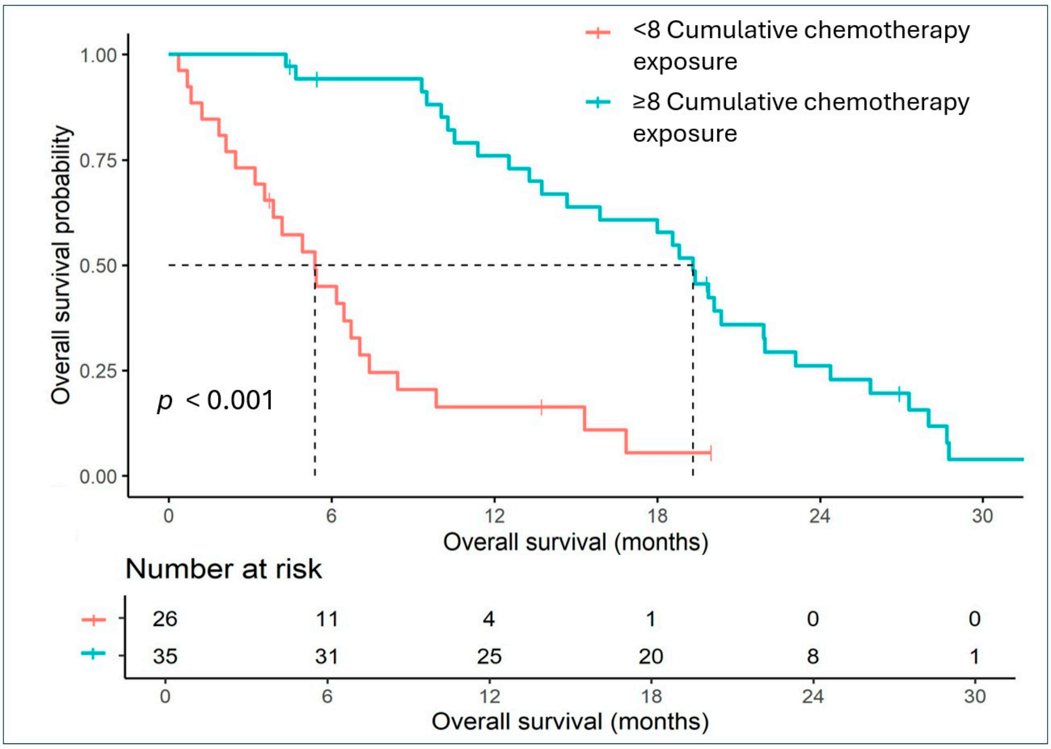

Supplementary Figure S1. Kaplan–Meier survival curves according to cumulative chemotherapy exposure after exclusion of patients who received GCD as first-line therapy (N = 61).

Supplementary Table S1. Treatment-course characteristics of the study cohort.

**Variable**	Overall (n = 86)
Median cumulative chemotherapy cycles (IQR)	7.0 [4.0, 12.0]
Patients with ≥1 dose reduction, n (%)	72 (83.7%)
**Number of chemotherapy regimens received, n (%)**	
1 regimen only	65 (75.5%)
2 regimens	17 (21.0%)
≥3 regimens	3 (3.5%)

Abbreviations: IQR, interquartile range. Cumulative chemotherapy cycles were calculated across all treatment lines. Dose reduction was defined as any reduction in chemotherapy dose during the treatment course.

Supplementary Table S2. Multivariable time-dependent Cox proportional hazards analysis of overall survival in the landmark cohort after adjustment for ECOG-PS (N = 66).

**Variables**	HR 95%CI	P value
Chemotherapy cycles (≥8 vs. <8)	0.44 (0.21 – 0.94)	0.033
Albumin, g/dL (≥ 3.5 vs. <3.5)	0.49 (0.24 – 0.99)	0.0498
ECOG-PS status (0 vs ≥1)	0.69 (0.20 – 2.50)	0.574
Metastasis (vs. locally advanced)	1.58 (0.81 – 3.10)	0.173
Total bilirubin, mg/dL (≥ 1.2 vs <1.2)	0.65 (0.30 – 1.37)	0.257
CA19-9, U/mL (≥100 vs. <100)	1.08 (0.54 – 2.13)	0.817
Cholangitis at baseline (yes vs. no)	0.73 (0.47 – 1.14)	0.170

Abbreviations: CA19-9, carbohydrate antigen 19-9; CI, confidence interval; ECOG-PS, Eastern Cooperative Oncology Group Performance Status; HR, hazard ratio; OS, overall survival.

Chemotherapy cycles were included as a time-dependent covariate in the Cox model.
